# Medical adherence to intranasal corticosteroids in adult patients^☆^

**DOI:** 10.1016/j.bjorl.2016.06.007

**Published:** 2016-07-20

**Authors:** Emre Ocak, Baran Acar, Deniz Kocaöz

**Affiliations:** Kecioren Training and Research Hospital, Department of Otorhinolaryngology, Ankara, Turkey

**Keywords:** Adherence, Allergic rhinitis, Intranasal corticosteroids, Treatment, Adesão, Rinite alérgica, Corticosteroides intranasais, Tratamento

## Abstract

**Introduction:**

The adherence to medical treatment in allergic rhinitis is poorly evaluated in clinical practice.

**Objectives:**

To evaluate adherence to intranasal corticosteroids in the treatment of allergic rhinitis patients.

**Methods:**

This prospective study was conducted on adult patients who were admitted to the outpatient clinic of the otolaryngology department tertiary hospital. Patients diagnosed with moderate to severe persistent AR and who had not used any nasal sprays were enrolled in the study. The patients were provided with mometasone furoate nasal sprays. On the 30th day, all participants filled out a questionnaire regarding the factors that may have influenced their adherence to the treatment. Afterwards, each patient filled out the Turkish-language-validated Morisky Medical Adherence Scale (MMAS-8) form. Each factor that may have affected adherence to the prescribed medication was evaluated according to the MMAS-8 score and all variables were analyzed statistically.

**Results:**

Fifty-nine adult patients with a mean age of 32.5 years (range 21–52 years) were included in the study. The mean overall MMAS-8 score was 3.64. Two factors were significantly related to low adherence: number of dependent children (*p* = 0.001) and benefit from the medication (*p* = 0.001). In addition, patients with higher education levels seemed to be more adherent than the rest of the group.

**Conclusion:**

Clinicians must keep in mind the factors related to non-adherence in order to achieve better treatment outcomes. Therefore, based on our results, patients must be informed that medications should be taken properly regardless of the benefit, and the treatment should be scheduled with respect to daily activities, particularly for patients caring for more than two children.

## Resumo

**Introdução:**

A adesão ao tratamento clínico de rinite alérgica é mal avaliada na prática clínica.

**Objetivos:**

Avaliar a adesão aos corticosteroides intranasais no tratamento de pacientes com rinite alérgica.

**Método:**

Este estudo prospectivo foi realizado em pacientes adultos admitidos no ambulatório do setor de otorrinolaringologia de um hospital terciário. Os pacientes diagnosticados com rinite alérgica moderada a persistente grave e que não haviam ainda usado spray nasal foram incluídos no estudo. Os pacientes receberam sprays nasais de furoato de mometasona. No 30° dia, todos os participantes preencheram um questionário sobre os fatores que podem ter influenciado a sua adesão ao tratamento. Depois disso, cada paciente preencheu o formulário da Escala de Adesão Clínica Morisky validado para a língua turca (MMAS-8). Cada fator que pode ter afetado a adesão à medicação prescrita foi avaliado de acordo com o escore de MMAS-8 e todas as variáveis foram analisadas estatisticamente.

**Resultados:**

Foram incluídos no estudo 59 pacientes adultos com média de 32,5 anos (variação de 21-52). O escore total médio de MMAS-8 foi de 3,64. Dois fatores foram significantemente relacionados com a baixa adesão: número de dependentes infantis (*p* = 0,001) e benefício da medicação (*p* = 0,001). Além disso, os pacientes com níveis de ensino mais elevados pareceram ser mais aderentes do que o restante do grupo.

**Conclusão:**

Os médicos devem estar cientes dos fatores relacionados à falta de adesão, a fim de alcançar melhores resultados do tratamento. Portanto, com base em nossos resultados, os pacientes devem ser informados de que os medicamentos devem ser usados adequadamente independentemente do benefício, e o tratamento deve ser programado com relação às atividades diárias, especialmente para os pacientes que cuidam de mais de dois filhos.

## Introduction

Adherence is defined as ‘the extent to which a person's behavior–taking medication, following a diet, and/or executing lifestyle changes – corresponds with agreed recommendations from a health care provider’.^1^ Non-adherence to prescribed medications has always attracted less attention in the treatment of disease. In particular, individuals who suffer from chronic diseases such as asthma, hypertension, diabetes *mellitus*, or Chronic Obstructive Pulmonary Disease (COPD) have certain difficulties with continuing medical treatment. This has been studied in several papers, and in one study regarding asthma patients, it was reported that less than half of prescribed medications were taken.^2^ Awareness of problems with adherence is as important as setting up the right treatment modality in such diseases. It is obvious that non-adherence will have a negative effect on long-term treatment results and obliviousness about this problem will lead to unnecessary therapies and incorrect decisions. Another study by Evans et al. indicated a discontinuation rate of 39% in hypertension.^3^ The rate is even higher in COPD patients, at 86% according to Penning-van Beest et al.^4^ These findings emphasize the importance of adherence to medical treatments.

The most commonly used scale for objectively evaluating adherence is the Morisky Medication Adherence Scale (MMAS), which is a generic, self-reported, medication-taking-behavior scale that was initially validated for hypertension but is used for a wide variety of medical conditions.^5^ The original version consisted of four items, and it was eventually updated to an eight-item scale called the MMAS-8 (Table 1).^6^ According to this scale, more points indicate lower adherence to treatment.

**Table 1:** MMAS-8 scale.

	Yes	No
1. Do you sometimes forget to take your pills?		
2. People sometimes miss taking their medications for reasons other than forgetting. Thinking over the past two weeks, were there any days when you did not take your medicine?		
3. Have you ever cut back or stopped taking medicine without telling your doctor because you felt worse when you took it?		
4. When you travel or leave home, do you sometimes forget to bring along your medicine?		
5. Did you take all your medicine yesterday?		
6. When you feel like your symptoms are under control do you sometimes stop taking your medicine?		
7. Taking medicine for everyday is a real inconvenience for some people. Do you ever fell hassled about sticking to your treatment plan?		
8. How often do you fell difficulty remembering to take all your medicine?		
		Never/rarely
		Once in a while
		Sometimes
		Usually
		All the time

Intranasal corticosteroids (ICS) are the mainstay medication for allergic rhinitis (AR) in the daily routine of outpatient otorhinolaryngology clinics. However, adherence to this treatment has not been well-studied. In the present study, we investigated adherence rates and factors that may lead to non-adherence in an adult AR population.

## Methods

### Study design and patient population

This prospective study was conducted on adult patients who were admitted to the outpatient clinic of the otolaryngology department at a tertiary hospital. Patients who were diagnosed with moderate to severe persistent AR according to the ARIA (allergic rhinitis and its impact on asthma) guidelines and who had not previously used any nasal sprays were enrolled.^7^ Informed consent was obtained from all patients before the study began. The institutional review board approved this study (IRB no. 665).

After a detailed clinical history and examination of the nasal cavity, sinuses, nasopharynx, and chest either with a telescope or X-rays, the diagnosis of AR was confirmed with evidence of specific IgE reactivity to allergens, determined on a Skin-Prick Test (SPT) and/or by the demonstration of serum-specific IgE. Patients with asthma, nasal septal deviation, a history of nasal spray use, acute/chronic sinusitis, a history of sinonasal surgery, or a history of sinonasal malignancy were excluded. After diagnosis, the patients were provided with mometasone furoate nasal spray at a dose of 256 μg per day, administered as two sprays per nostril once daily in the morning for 30 days. All patients were given written standardized instructions on how to use the medication. At the end of the therapy, all participants were seen for a control examination and were asked to fill out an 11 item questionnaire regarding the factors that may have influenced their adherence to the treatment (Table 2). After the questionnaire, each patient filled out the Turkish-language-validated MMAS-8 form and the scores were calculated by another physician who was blind to the clinical data.^8^ MMAS-8 is a practical scale comprised of 8 yes/no questions regarding patient's adherence to medical treatment. As described above higher scores mean less adherence to treatment. Each factor that may have affected adherence to the prescribed medication was evaluated according to the MMAS-8 score and all variables were analyzed statistically.

**Table 2:** Factors related to adherence level.

		*n*	MMAS-8 score	*p*
Age	≤35	30	3.40	0.413
	>35	29	3.89	

Gender	Female	25	3.28	0.387
	Male	34	3.96	

Maritial status	Single	19	3.50	0.827
	Married	40	3.71	

Multidrug use	Yes	26	3.75	0.987
	No	33	3.42	

Comorbidities	Yes	20	3.68	0.778
	No	39	3.55	

Daily working hours	≤6 h	33	4.00	0.327
	>6 h	26	3.41	

Children	≤2	35	3.13	0.001^a^
	>2	24	6.87	

Side effects	Yes	12	5.07	0.150
	No	47	3.23	

Benefit from the drug	Yes	38	2.89	0.001^a^
	No	21	6.90	

Abroad days per month	≤5 days	36	3.24	0.081
	>5 days	23	4.92	

a Statistically significant.

### Statistical analysis

Statistical analysis was performed using the Statistical Package for the Social Sciences version 15.0 (SPSS, Inc., Chicago, IL, USA). The significance of the difference between groups in terms of median values was determined with the Kruskal–Wallis test. A *p*-value of <0.05 was considered significant.

## Results

From 82 patients who were diagnosed as AR in our outpatient clinic, 59 patients (32 female and 27 male) with a mean age of 32.5 years (range 21–52) who met the criteria and come back for assessment at 30 days were included in the study. The remainder 23 patients were excluded. Forty-two patients of the study group had a positive SPT and 23 had specific IgE reactivity to allergens (6 patients had both positive SPT and specific IgE reactivity). The mean overall MMAS-8 score was 3.64. The factors that may affect adherence and the related MMAS-8 scores are summarized in Table 2. When the scores were evaluated, patients living with more than two dependent children, who did not benefit from the medication, who experienced side effects, or who traveled more than 5 days per month had higher scores for poor adherence (6.87, 6.90, 5.07, and 4.92 points, respectively). When all questions were evaluated from the statistical point of view, two factors seemed to be significantly related to low adherence: number of dependent children (*p* = 0.001) and benefit from the medication (*p* = 0.001). In our study, patients with more than two dependent children and who thought that the medication did not work were more likely to discontinue their therapy. On the other hand, patients who benefited from the medication, those who were living with less than two dependent children, and those who had fewer side effects were more adherent to the therapy according to MMAS-8 scores (2.89, 3.13, and 3.23 points, respectively). Education status was another important factor. Patients with higher education levels seemed to be more adherent than the rest of the group (Table 3).

**Table 3:** MMAS-8 scores according to education level.

Education^a^	MMAS-8
Primary school	3.11
Middle school	6.71
High school	5.63
University/postgraduate	2.43^b^

a Education levels are shown according to the Turkish education system.

b Statistically significant according to nonparametric tests.

## Discussion

The importance of adherence to medical therapy is an incontestable element that may affect treatment outcomes. It is beyond doubt that non-adherence will cause prolonged duration of treatment, dissatisfaction of patients, low quality of life, and unnecessary financial burdens for governments. A report stated that more than half of patients with chronic diseases fail to continue prescribed medication.^9^ Adherence is an underestimated factor in the treatment protocols for various diseases, but the number of studies investigating this phenomenon has gradually increased in the last decade. In a study by Christensen, non-adherence to medical treatment regimens was reported to range between 20% and 40% in acute illness and 30%–60% in chronic diseases. Moreover, discontinuity rates were as high as 80% for preventive treatments.^10^ There are numerous determinants of adherence. In general, these can be divided into modifiable and non-modifiable factors, such as the type of prescribed medication, the physician–patient relationship, the cost and the disease itself as stated by Osterberg et al.^11^

Although investigations about medical adherence have attracted much attention lately, adherence to ICS in AR is less well studied. A few reports were published about the role of patient preference, knowledge level and willingness to adhere to the therapy.^12^, ^13^, ^14^

Based on this background, we investigated the variables that may be related to medical adherence to ICS in AR. Our findings suggested that the number of children dependent on the patient and the feeling of receiving a benefit from the drug were the two most significant factors relevant to adherence. Providing benefit from the drug is a subjective term. Our data demonstrates that it is an important factor and the patients who were in the feeling that the medication is working, seemed to be more adherent to the treatment. Thus, we suggest scheduling the medication plan with respect to daily activities, particularly for patients caring for more than two children. It is also important to remind the patient to take the prescribed medications properly, regardless of the benefit. In order to avoid non-adherence due to the above-mentioned factors, it is essential to reexamine the patient at regular intervals and to change the medicine if necessary.

It is important to note that neither efficiency nor benefit from the treatment was evaluated in this study. Therefore, a homogenous study group was formed in terms of gender, age, and severity of disease.

Limitations of this study may be the number of participants, the possibility of interaction between the factors, and the follow-up period. However, as mentioned above, meticulous effort was made to prevent heterogeneity in the study population. On the other hand, a precise and independent evaluation of each factor requires an excessively homogenous study group, which in practice would reduce the number of patients. Another factor is the lack of multivariate statistical analyses due to the limited number of participants. Thus, further studies with larger groups and longer follow-up periods are needed to perform a more accurate evaluation of adherence in AR.

## Conclusion

The treatment of a disease is a multifactorial process involving the appropriate drugs, timing, the patient's general status, and the healthcare system. After the diagnosis is made and while the clinician is setting up the treatment regime, it must be kept in mind that non-adherence will frustrate all efforts. Therefore, we suggest keeping in mind the factors related to non-adherence when providing information about prescribed medications.

## Conflicts of interest

The authors declare no conflicts of interest.
